# IgY antibodies against Ebola virus possess post-exposure protection in a murine pseudovirus challenge model and excellent thermostability

**DOI:** 10.1371/journal.pntd.0008403

**Published:** 2021-03-12

**Authors:** Yuan Zhang, Yanqiu Wei, Yunlong Li, Xuan Wang, Yang Liu, Deyu Tian, Xiaojuan Jia, Rui Gong, Wenjun Liu, Limin Yang

**Affiliations:** 1 Key Laboratory of Pathogenic Microbiology and Immunology, Institute of Microbiology, Chinese Academy of Sciences, Beijing, China; 2 Anhui University, Hefei, Anhui, China; 3 The Biological Safety level-3 (BSL-3) Laboratory of Institute of Microbiology, Chinese Academy of Sciences, Beijing, China; 4 CAS Key Laboratory of Special Pathogens and Biosafety, Wuhan Institute of Virology, Center for Biosafety Mega-Science, Chinese Academy of Sciences, Wuhan, Hubei, China; 5 Institute of Microbiology, Center for Biosafety Mega-Science, Chinese Academy of Sciences, Beijing, China; University of Texas Medical Branch / Galveston National Laboratory, UNITED STATES

## Abstract

Ebola virus (EBOV) is one of the most virulent pathogens that causes hemorrhagic fever and displays high mortality rates and low prognosis rates in both humans and nonhuman primates. The post-exposure antibody therapies to prevent EBOV infection are considered effective as of yet. However, owing to the poor thermal stability of mammalian antibodies, their application in the tropics has remained limited. Therefore, a thermostable therapeutic antibody against EBOV was developed modelled on the poultry(chicken) immunoglobulin Y (IgY). The IgY antibodies retaining their neutralising activity at 25°C for one year, displayed excellent thermal stability, opposed to conventional polyclonal antibodies (pAbs) or monoclonal antibodies (mAbs). Laying hens were immunised with a variety of EBOV vaccine candidates and it was confirmed that VSVΔG/EBOVGP encoding the EBOV glycoprotein could induce high titer neutralising antibodies against EBOV. The therapeutic efficacy of immune IgY antibodies *in vivo* was evaluated in the newborn Balb/c mice who have been challenged with the VSVΔG/EBOVGP model. Mice that have been challenged with a lethal dose of the pseudovirus were treated 2 or 24 h post-infection with different doses of anti-EBOV IgY. The group receiving a high dose of 10^6^ NAU/kg (neutralising antibody units/kilogram) showed complete protection with no symptoms of a disease, while the low-dose group was only partially protected. Conversely, all mice receiving naive IgY died within 10 days. In conclusion, the anti-EBOV IgY exhibits excellent thermostability and protective efficacy. Anti-EBOV IgY shows a lot of promise in entering the realm of efficient Ebola virus treatment regimens.

## Author summary

Despite the amount of efficient Ebola virus therapeutic antibodies reported in recent years, their application in tropical endemic areas has remained limited due to the low thermal stability of mammalian antibodies. A highly thermostable therapeutic polyclonal antibody against EBOV was developed based on chicken immunoglobulin Y (IgY). The EBOV specific IgY antibodies displayed excellent thermal stability, retaining their neutralising activity at 25°C for one year. The newborn mice receiving the passive transfer of IgY achieved complete protection against a lethal dose of virus challenge proving that the anti-EBOV IgY provides a promising recourse to solve some of the current clinical application hindrances of Ebola antibody-based treatments in Africa due to thermal stability.

## Introduction

Ebola virus (EBOV) belongs to the Filoviridae family and is the known cause of severe hemorrhagic fever in humans and nonhuman primates (NHPs) [1]. Since the first epidemic in Zaire (presently known as the Democratic Republic of the Congo, DRC) and Sudan in 1976 [2], recurring outbreaks still occur in Africa, with a case-fatality rate averaging 25–90% [3, 4]. The most significant Ebola outbreak to date has happened in West Africa from 2014 to 2016, infecting more than 28,000 people, causing 11,323 fatalities [5, 6]. Imported cases have also been reported as a result of travelling from Africa. The world’s second largest 2018–2020 Ebola outbreak on record was incredibly challenging, and prompted the WHO to declare that outbreak a public health emergency of international concern [7, 8]. Pandemic potential, high mortality and high infectivity make EBOV a Class A pathogen that constitutes a severe threat in the face of public health.

The intermittent and the continuous outbreak of the Ebola virus disease (EVD) is a significant risk to public health and has kindled extensive research on vaccines and antiviral drugs. The most effective strategy, as of yet, to prevent and treat EVD, is antibody immunotherapy [9–13]. However, antibody treatment failed to show any promise at the beginning. Although the antibody KZ52 had high neutralising activity but was unable to play a role in NHPs [14]. In 2012, it was discovered that convalescent plasma could protect NHPs from lethal doses of EBOV infection, and academic research in antibody therapy strategy was rekindled [15]. The researchers tried to mix multiple mAbs against EBOV glycoprotein (GP) to form ZMapp (2G4, 4G7, 13C6), a new antibody combination based on a cocktail therapy[16, 17]. ZMapp was shown to have excellent protective effects in NHPs and patients and has greatly extended the window period of administration[18, 19]. Furthermore, the emergence of ZMapp has brought about the peak of antibody treatment research [20]. The FDA approved REGN-EB3, a mixture of three mAbs, as the first approved treatment for Zaire EBOV infection in adult and pediatric patients[21]. mAb114 is a single mAb isolated from a survivor of the 1995 Kikwit outbreak that targets the receptor-binding domain of Ebola virus glycoprotein, which prevents mortality in rhesus macaques treated after lethal challenge with Zaire ebolavirus[22, 23]. MAb114 (Ebanga) was superior to ZMapp in reducing mortality from EVD during disease outbreaks [24], which was also the second Ebola drug approved by the FDA. Nowadays, antibody therapy is a promising approach to control EVD. Furthermore, anti-EBOV equine sera and ovine sera were recently confirmed to be able to efficiently protect rodent models against EBOV challenge [25–27]. These studies suggest that EBOV-specific antibodies from different species are expected to be developed as therapeutic agents.

Although EBOV therapeutic antibody has inspiring application prospects, application limitations still loom large. Firstly, the limited sources and security of convalescent plasma have hampered its global application. Secondly, mAbs have long preparation cycles and expensive production costs. Furthermore, they all require strict transportation and storage conditions. However, the Ebola outbreak mainly occurred in the temperate and more impoverished African regions, effectively limiting the current application of antibodies. Poultry-derived IgY provides a secure and efficient alternative strategy for producing safe and affordable antibodies. IgY antibodies, the predominant serum immunoglobulin in birds, reptiles, and amphibians, are transferred from the serum of females to the egg yolk [28], where they offer passive immunity to embryos and neonates. As a potential therapeutic antibody, IgY has many advantages over mammalian IgG due to its structural and immunological properties [29]. It displays excellent stability under various physicochemical conditions and incurs lower manufacturing costs. It is unable to bind to mammalian Fc receptors or complement components [30], therefore effectively circumventing potential antibody-dependent infection enhancement (ADE) and adverse immune reactions. Eggs can be used to produce large amounts of yolk antibodies in a shorter period of time and do not display adverse effects on animals. In recent years, the antibody therapy strategy based on IgY has been widely recognised, and many promising results have been documented in the treatment of influenza virus[19, 31], dengue virus [32], zika virus [33], hantavirus [34], SARS virus [35], Rotavirus and norovirus [36] infections, etc.

This study aimed to produce an anti-EBOV IgY antibody and assess its antiviral efficiency. The IgY antibody was harvested from laying hens immunised with recombinant vesicular stomatitis virus vector encoding EBOV GP (VSVΔG/EBOVGP). The protective efficiency of the resulting IgY antibody against EBOV was evaluated by Enzyme-linked immunosorbent assay (ELISA), pseudotyped virus neutralisation assay, and a mouse challenge model. The results prove that the IgY antibody has excellent thermal stability and is efficient against lethal challenge with VSVΔG/EBOVGP in newborn mice. Our results suggest that the potent IgY warrants further development as prophylactic and therapeutic reagents for EVD.

## Results

### Preparation of immunogens

**Fig 1:**
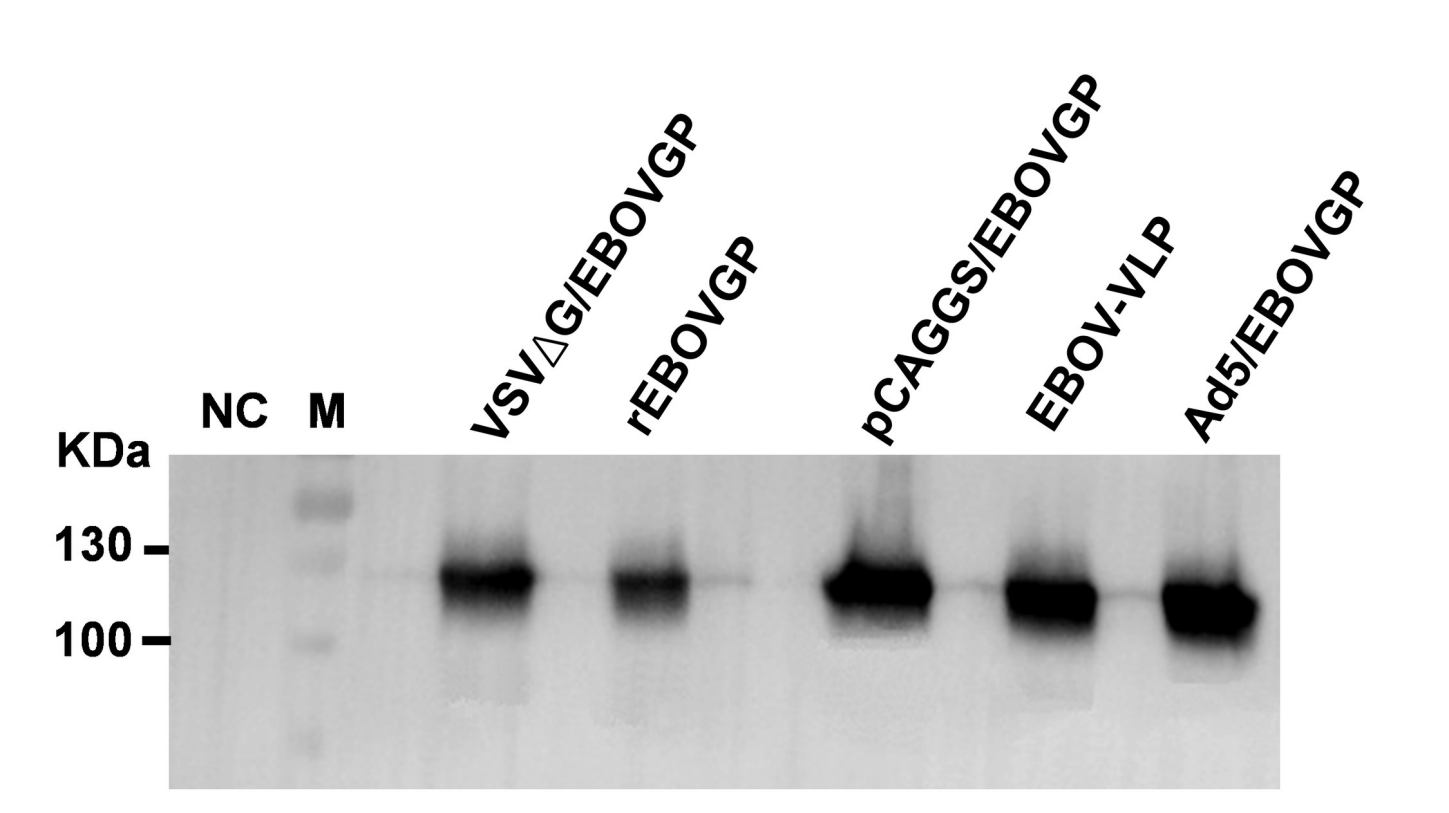
Western blot analysis of EBOV immunogens. Subunit (rEBOVGP, EBOV-VLP), vector (VSVΔG/EBOVGP, Ad5/EBOVGP) immunogens and 293T cell lysates transfected with plasmid expressing EBOV GP (pCAGGS/EBOVGP) were separated by SDS–PAGE under reducing conditions, transferred to PVDF membrane, and detected using EBOV GP1-specific mAb. Lane NC is 293T cell lysates transfected with pCAGGS plasmid.

Vaccine-elicited neutralising antibodies (NAbs) are associated with protection against Filoviridae family mediated disease [37, 38]. To obtain the most potent anti-EBOV antibody, several EBOV immunogens based on multiple different platforms were prepared, including DNA vaccine (pCAGGS/EBOVGP), recombinant protein (rEBOVGP) or virus-like particle (EBOV-VLP) sub-unit vaccines, and two viral vector vaccines (VSVΔG/EBOVGP, Ad5/EBOVGP). Western blot confirmed that these immunogens could express or contain EBOV GP that can induce NAbs in animals (Fig 1). Due to the differences in humoral immune responses induced by different vaccines, the most suitable immunogen for IgY antibody production needed to be isolated.

### Immunogens elicited high titer of anti-EBOV IgY

**Fig 2:**
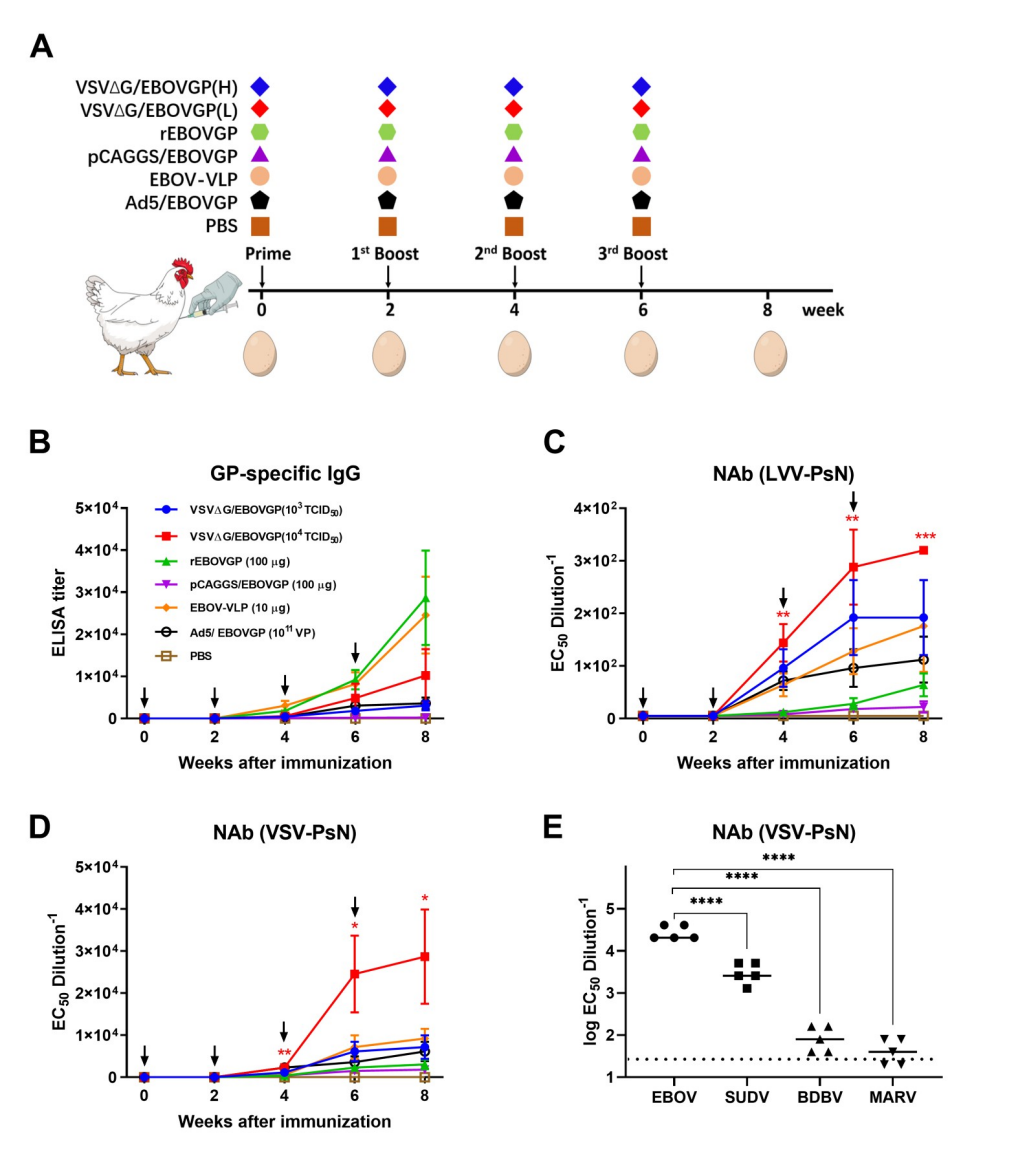
Immunogenicity of different EBOV vaccine candidates in laying hens. (A) Seven groups of laying hens (5-month-old, n = 5 per group) were inoculated with VSVΔG/EBOVGP, rEBOVGP, pCAGGS/EBOVGP, EBOV-VLP, Ad5/EBOVGP or PBS as control, respectively, through i.m. route at 0, 2, 4, 6 weeks. The eggs were collected at 0, 2, 4, 6, 8 weeks respectively. The yolk IgY were purified and used to test the GP-specific ELISA antibody titers (B) and NAb titers (C, D) by LVV-PsN and VSV-PsN assay. A two-way analysis of variance (ANOVA) with Tukey’s multiple-comparison tests was performed to statistically analyse significant differences among different immunogen groups. Significant differences were observed between VSVΔG/EBOVGP and PBS at 4, 6, and 8 weeks post immunisation. The NAb titers of IgY antibodies collected at week 8 against EBOV, SUDV, BDBV and MARV were evaluated using VSV-PsN respectively (E). A one-way ANOVA test was performed to indicate statistical significance among different NAb test groups. The black arrows in b, c and d represent the immunisation time point. The dashed line in e indicates the detection limit. Data are shown as means ± SEM. In all panels with *P* values: *P < 0.05; **P < 0.01; ***P < 0.001; ****P < 0.0001.

To obtain high titer EBOV NAbs, 5-month-old laying hens were vaccinated with five different immunogens, including 10^3^ or 10^4^ TCID_50_ VSVΔG/EBOVGP, 100 μg rEBOVGP, 100 μg pCAGGS/EBOVGP, 10 μg EBOV-VLP, and 10^11^ virus particles (vp) Ad5/EBOVGP (Fig 2A). Thirty-five laying hens were randomly allocated into seven groups, and were immunised four times with each immunogen or PBS control intramuscularly (i.m.) through a 14-day interval. Eggs were collected at 0, 2, 4, 6, 8 weeks, and IgY antibodies were purified from egg yolk for ELISA and NAbs tests. Both titers in all groups were gradually increased after the first immunisation. The results showed that all immunogens, besides the DNA vaccine, induced potent Gp-specific ELISA antibodies (Fig 2D). As for the NAbs, the geometric mean titers (GMTs) in the 10^4^ TCID50 VSVΔG/EBOVGP group reached 1:40960 (VSV pseudoneutralisation, VSV-PsN) and 1:320 (lentiviral vectors pseudo-neutralisation, LVV-PsN) after the third boost, which was significantly higher than the other groups (Fig 2B and 2C). VSVΔG/EBOVGP induced the strongest NAb titers than the other immunogens. In contrast, the GP-specific ELISA and NAb titers were not detected in the negative control group. Besides, to determine the correlation between the immune dosage and antibody level, two dosages of VSVΔG/EBOVGP were used to immunise laying hens. 10^4^ TCID50 VSVΔG/EBOVGP induced stronger NAbs titer compared to 10^3^ TCID_50_ VSVΔG/EBOVGP, suggesting that the immunisation dose was crucial for obtaining higher titers of NAb. In order to circumvent the possible interference of the antibody generated by the VSV vector backbone, two PsN assays, VSV-PsN and LVV-PsN were used, and the two assays yielded consistent results. The IgY antibody was subsequently assessed for cross-protection against different filovirus species. The NAb titers of Sudan virus (SUDV), Bundibugyo virus (BDBV) and Marburg virus (MARV) were also tested using VSV-PsN assay. The results showed that the IgY antibody has a particular cross-neutralising activity with SUDV, but has very low NAb titers for BDBV and MARV, suggesting that the application of IgY prepared this time is restricted to emergency prevention of EBOV and SUDV infection (Fig 2E).

### Purification and characterisation of IgY

**Fig 3:**
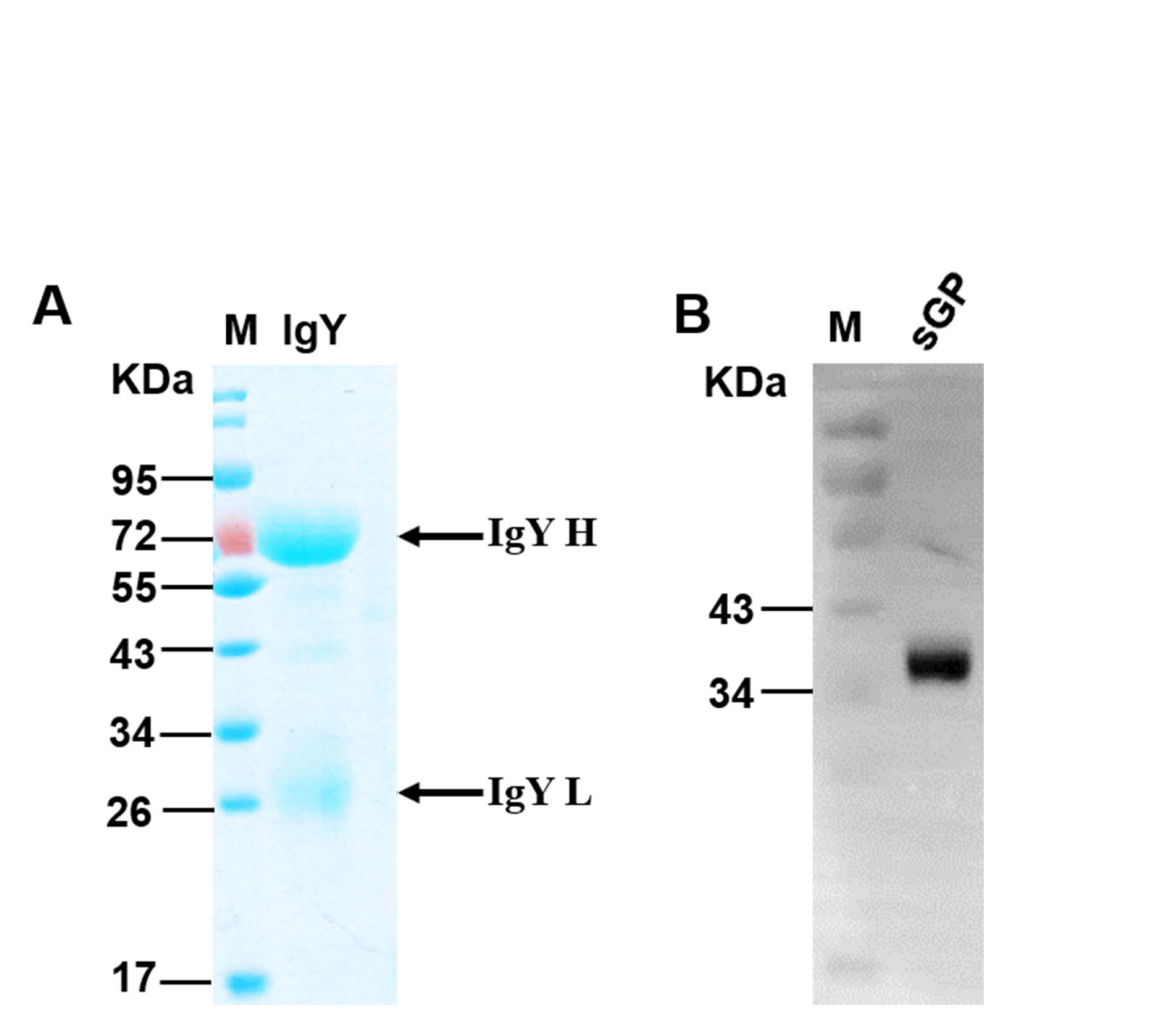
Purity and immunogenicity analysis of anti-EBOV IgY antibody. (A) Purified IgY isolated from the VSVΔG/EBOVGP vaccination group was analysed by SDS-PAGE. (B) Recombinant sGP expressed in *E*. *coli* was analysed by Western blot with anti-EBOV IgY. Recombinant sGP protein isolated by SDS-PAGE was incubated with EBOV specific IgY, then specific binding bands of sGP were detected by HRP-conjugated mouse anti-IgY mAb.

After the four immunisations with 10^4^ TCID50 VSVΔG/EBOVGP, IgY antibodies were purified from these collected eggs and then verified by SDS-PAGE (Fig 3A) and Western blot (Fig 3B) assays. SDS-PAGE results showed that two bands with molecular weights of 66 and 22 kDa appeared, representing the heavy chain and light chain of IgY, respectively. The purified IgY antibodies exhibited EBOV GP-specific immunoreactivity to EBOV sGP expressed in *E*. *coli* expression system when tested with Western blot. Besides, about 50 mg of IgY antibodies with a purity greater than 95% can be yielded from an egg.

### Passive transfer of IgY protect newborn BALB/c mice from lethal pseudovirus challenge

**Fig 4:**
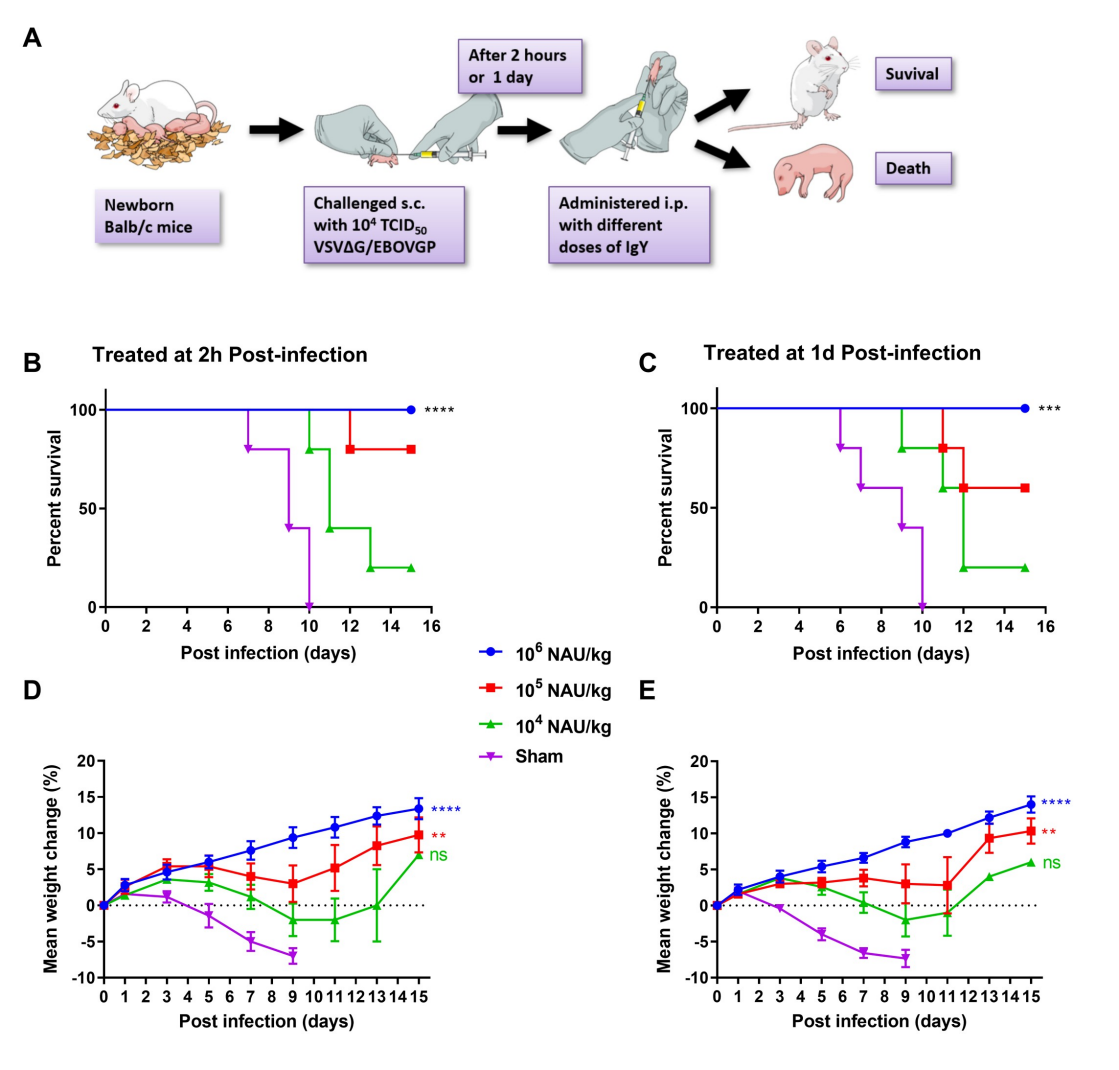
Passive protection of anti-EBOV IgY in newborn Balb/c mice pseudovirus challenge model. Experimental scheme (A). Eight groups of newborn Balb/c mice (3-day-old, n = 5 per group) were challenged s.c. with 10^4^ TCID50 VSVΔG/EBOVGP, after 2 h or 1 day, each mouse was given different doses of IgY or naïve IgY via i.p. route twice daily over 3 days. The survival rates (B, C) and body weight changes (D, E) were monitored and recorded daily. Survival data were analysed by two-sided log-rank test. Weight change was analysed by Tukey–Kramer multiple comparison test. Data are shown as means ± SEM. In all panels with P values: no significant difference (ns) *P* > 0.05; *P < 0.05; **P < 0.01; ***P < 0.001; ****P < 0.0001.

To determine the protective efficiency of the anti-EBOV IgY antibodies against EBOV, passive protection experiment was performed in newborn BALB/c mice (Within 3 days after birth). Forty newborn BALB/c mice were allocated into eight groups, which were challenged subcutaneously (s.c.) with 10^4^ TCID50 VSVΔG/EBOVGP. Two hours or 1-day post-infection (dpi), each mouse was adoptively transferred with IgY twice daily for 3 days, and the control group mice treated with naive IgY (Fig 4A). To determine the correlation between the transferred IgY dosage and therapeutic efficacy, three different dosages with 10^4^, 10^5^, or 10^6^ NAU/kg (NAb units/kilogram, VSV-PsN) were intraperitoneally (i.p.) transferred to six groups of challenged mice, respectively. The results showed that all the mice receiving 10^6^ NAU/kg IgY were healthy with the gradually increasing bodyweights and without any clinical symptoms within 15 dpi (Fig 4B and 4C). However, all the control mice died during 6–10 dpi, and the bodyweights began to plummet from the second day until death. In the other two lower dose administration groups, mice failed to harness full protection and displayed bodyweight losses after 3 dpi. Only one mouse receiving 10^4^ NAU/kg IgY survived to day 15, and the mice of two timepoints treatment groups increased 7% or 6% of body weight with a mean time to death (MTD) of 11.25 or 11 dpi (Fig 4D and 4E). The situation has improved when the injection amount was 10^5^ NAU/kg. 4/5 mice receiving IgY 2 h post-infection survived to day 15 with an average weight increase of 9.8% and an MTD of 12 dpi. Another group, 3/5 mice receiving IgY 1 dpi survived to day 15 with an average body weight increase of 10.3% and an MTD of 11.5 dpi. These data indicate that anti-EBOV IgY conferred complete passive protection to newborn mice against a high concentration of VSV vector-based EBOV recombinant chimeric virus challenge. Administrating high-concentration IgY immediately after infection or even one day later can prevent mice from dying due to infection, suggesting that anti-EBOV IgY can be used as an emergency prevention and treatment measure after exposure to EBOV. The new challenge model, which can be developed in BSL2 laboratory instead of live EBOV operation, was established for the first time. This model is only limited to mice born within three days, and mice born after three days will not die.

### Bioavailability of IgY in guinea pigs

**Fig 5:**
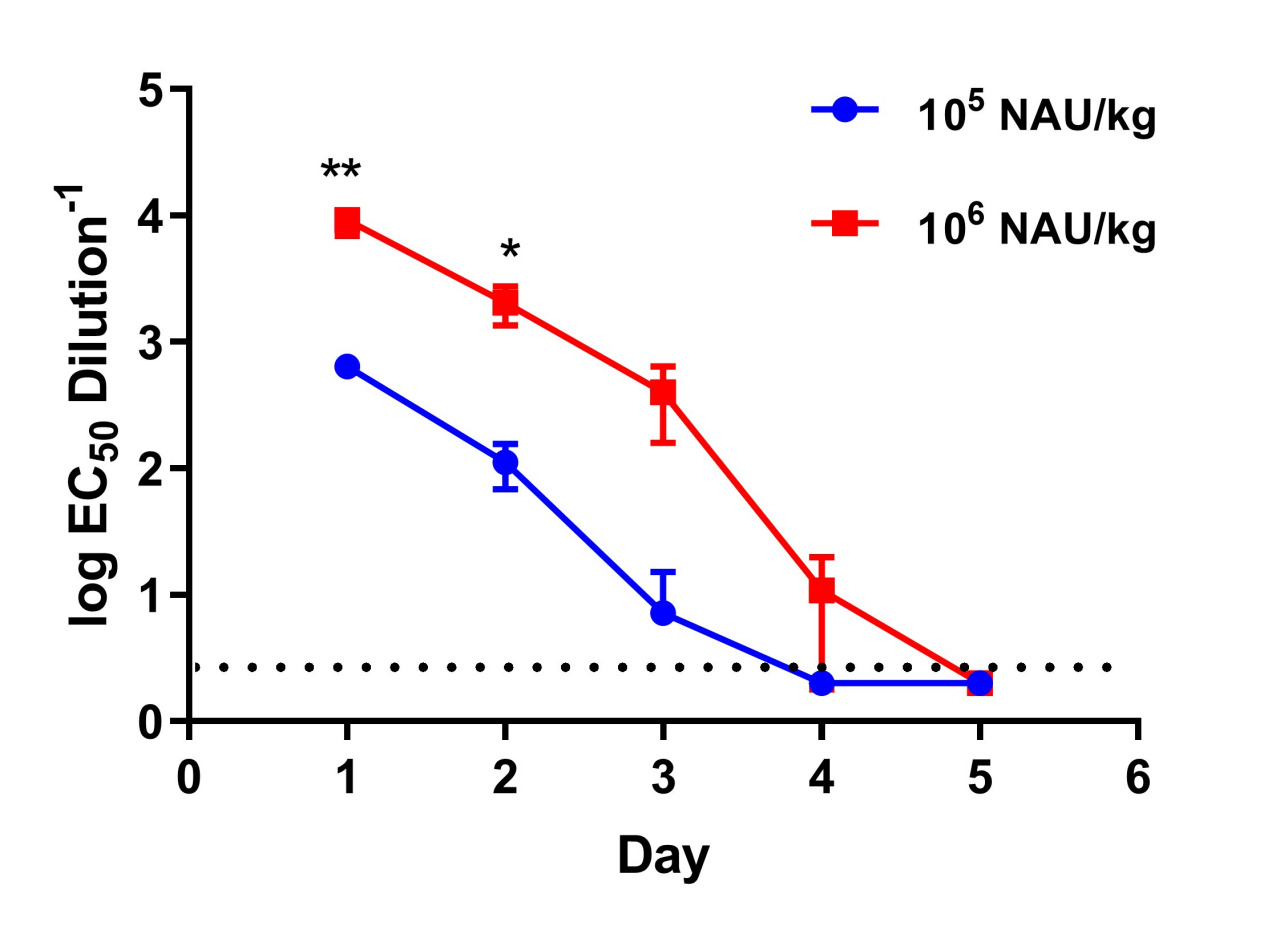
Bioavailability of anti-EBOV IgY in guinea pigs. Two Groups of guinea pigs (12-week-old, n = 3 per group) were injected s.c. with 10^6^ NAU/kg or 10^5^ NAU/kg IgY. Sera NAb titers were tested daily by VSV-PsN assay. A two-way ANOVA with Tukey’s multiple-comparison tests were performed to analyse statistically significant differences between the two different dose groups. Significant differences were observed at 1and 2 days post-injection (*P < 0.05; **P < 0.01). The dashed line in e indicates the detection limit. Data are shown as means ± SEM.

To further confirm the protective efficacy of the anti-EBOV IgY, the metabolic level of antibodies in the body needed to be evaluated. The bioavailability of IgY *in vivo* was assessed, two groups of 12-week-old female guinea pigs received s.c. injection with 10^5^ or 10^6^ NAU/kg IgY, respectively. Sera were collected daily and tested by means of a VSV-PsN assay within six days after antibodies injection. The NAb titers in the guinea pig sera reached a high level, followed by a decrease in antibody level and fell below the detection limit on the third (low dosage group) and fourth days (high dosage group), respectively (Fig 5). These results suggest that passive transfer of IgY can provide guinea pigs with 2–3 days of protection and that higher doses of antibodies can provide longer protection time.

### Thermal stability analysis of antibodies

**Fig 6:**
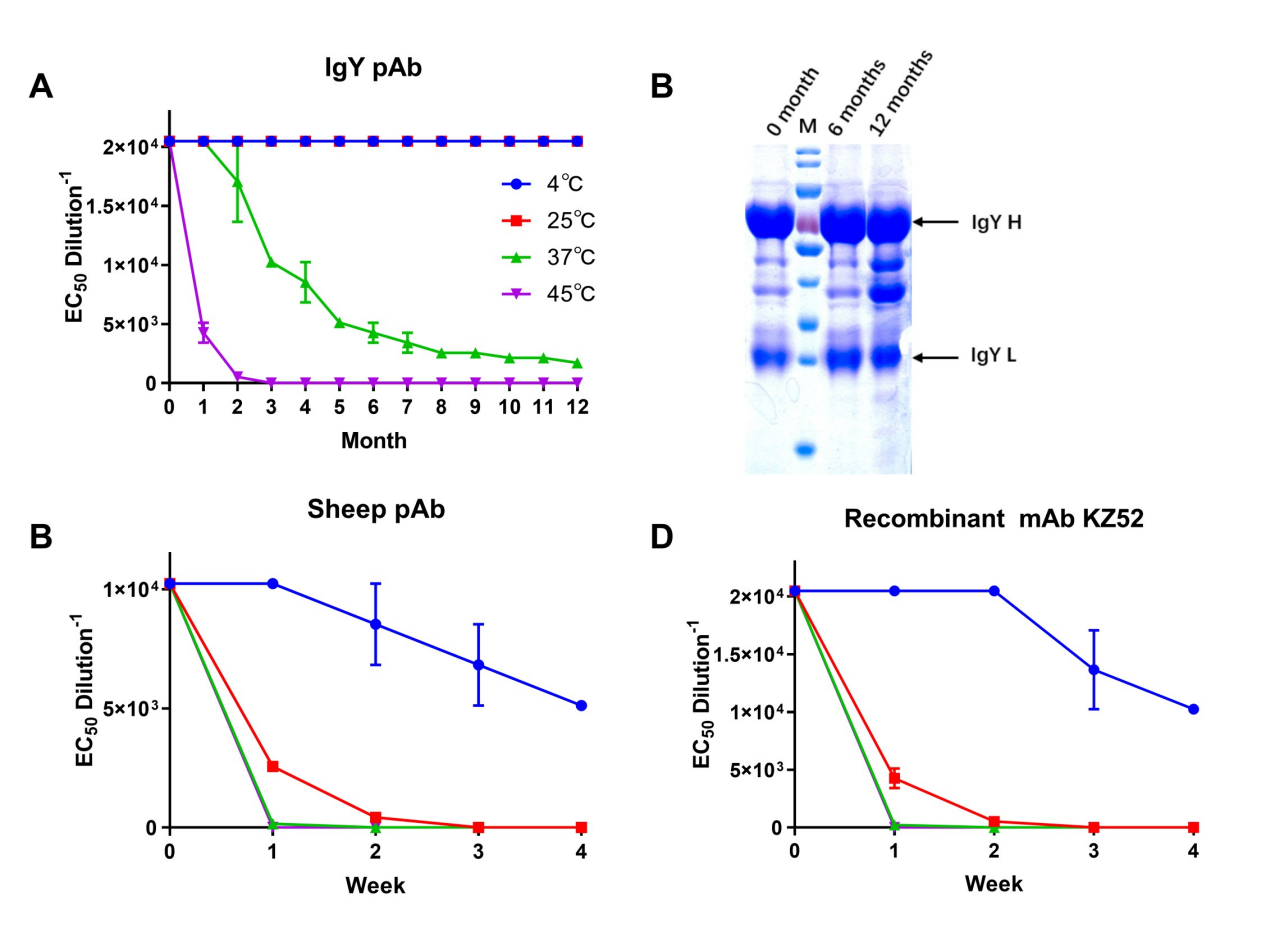
Thermal stability analysis of IgY pAb, sheep pAb and recombinant mAb against EBOV. Three different types of antibody solutions were placed under different temperature conditions (4°C, 25°C, 37°C, and 45°C) for 1 year or 4 weeks and NAb titers were detected per month (A) or week (C, D). The IgY stored at 25°C for 0, 6 and 12 months were analysed by SDS-PAGE (B). Data are shown as means ± SEM.

Considering that Ebola hemorrhagic fever mainly occurs in tropical regions, the thermal stability of antibody-based antiviral reagents is crucial for practical applications. The purified and filtered sterilised IgY, sheep pAb (Abcam, UK) and recombinant mAb KZ52 (IBT Bioservices, USA) solutions were stored in four different temperature environments, at 4°C, 25°C, 37°C, and 45°C. The NAb titers of IgY were subsequently tested every month for one year. The results showed that the IgY NAb titers displayed no significant change at 4°C and 25°C within a year. However, the antibody titer stored at 37°C gradually decreased from the second month, and only about 50% of the antibody activity remained at the third month. In contrast, the activity of the IgY stored at 45°C was lost faster, and the NAbs titer could not be measured by the second month (Fig 6A). The monthly samples were stored at -20°C, and finally, IgY helds at 25°C for 0, 6, and 12 months were verified by SDS–PAGE. After IgY was placed for 1 year, the results of SDS-PAGE showed no significant protein degradation, and only some extra bands appeared (Fig 6B), but the NAb titers were not significantly affected, which also proved that the IgY has excellent stability at room temperature. Compared with IgY, mammalian-derived antibodies exhibit relatively low thermal instability. Samples at different temperatures were taken every week for verification, and the results showed that the titers of antibody at different temperatures were significantly reduced, and both sheep pAb and KZ52 lost their activity within two weeks under conditions of high temperature (Fig 6C and 6D). These results proved that the anti-EBOV IgY has excellent thermal stability and can be stored at room temperature (RT) for up to one year. It can retain momentary activity even at a temperature of 45°C. It is suggested that, compared with mammalian-derived antibodies, this anti-EBOV IgY is more suited as an emergency prevention/treatment reagent in endemic tropical regions.

## Discussion

Ebola hemorrhagic fever is associated with high mortality in humans and rapid contamination among populations having poor medical facilities and infrastructure. Antibody therapy is the sole option for EBOV-infected patients in those areas. An ideal therapeutic antibody should be highly efficient, practical, and affordable to the masses. Previous strategies to develop an antibody against EBOV were derived from mammals, these antibody reagents have proven excellent therapeutic effects, and higher stability is obtained partially through antibody modification or lyophilised technology including FDA-approved lyophilised therapeutic antibody drugs Ebanga [24], but the problem of generally poor thermal stability of mammalian antibodies still lingers. [12, 15, 20, 23, 25–27, 39–43]. Given that EVD mainly occurs in Africa, especially in some impoverished and remote areas, due to the temperate climate and lack of cold chain transportation, the clinical application of these antibodies may be compromised. Moreover, the sooner people receive treatment post-exposure to EBOV, the higher the survival rate. Therefore developing an EBOV therapeutic antibody with high-efficiency protection and good thermal stability has been prioritised. In recent years, therapeutic antibodies based on avian IgY have been the subject of intense scrutiny, and a variety of pathogen-specific antibodies based on IgY have proved to have excellent safety and therapeutic effects [31–33, 35, 36, 44, 45]. Compared with mammalian antibodies, avian antibodies have several distinct advantages. First of all, IgY has higher target specificity, greater binding avidity and longer circulating half-life, which could potentially increase its efficacy against infections; Secondly, IgY does not react with complement systems, so the risk of causing inflammation *in vivo* is reduced[46]. In addition, previous studies have shown that EVD has an ADE effect, depending on the cross-linking of complexes of virus-antibody or virus-activated complement components through interaction with cellular molecules such as Fc receptors or complement receptors. This could potentially lead to enhanced infection of susceptible cells, which could enhance EBOV infection by human antibodies[47]. Blocking of Fc-receptors reaction through mutating the Fc fragment can abolish ADE, and can effectively enhance the protective efficacy of the EBOV antibody [47]. As IgY does not bind to mammalian Fc receptors, it can perfectly circumvent the EBOV ADE effect caused by antibodies [32, 33]. Furthermore, one hen can produce up to 22 grams of IgY antibody a year, which means that the antibody production of two hens is equivalent to that of one horse, and egg production has already been scaled up, making large-scale and low-cost IgY production feasible [48]. The cheaper antibody drugs are also more suitable for application in Africa. In addition, compared with antibodies derived from mammalian serum, IgY is derived from eggs, and therefore abide more with animal protection principles and address ethical concerns. Finally, IgY displays excellent thermal stability, and can even be stored at RT for more than 6 months and at 37°C for more than 1 month, while maintaining its activity [48]. There are still some problems to be solved in the conversion of IgY antibody products into clinical practice in humans. The immunogenicity of IgY has been previously verified in pigs and mice [49, 50]. These data suggest that IgY is antigenic and although the biochemical properties of this antibody molecule do not facilitate considerable binding to mammalian Fc receptors, serum sickness is a theoretical possibility if IgY is administered in large amounts. Therefore, for treatments that require high doses, multiple injections are required. In addition, the polyclonal IgY is poorly defined, Antiviral potential requires rigorous evaluation of multiple methods.

Although multiple EBOV vaccine platforms have been established, several vaccine candidates showed excellent protective efficacy, but a systematic comparison of the humoral immunity levels of different candidates on an animal model has not been addressed. Therefore, five different vaccine candidates were tested in laying hens for eliciting yolk IgY level. It was demonstrated that the VSV vector-based EBOV vaccine could induce laying hens to produce high-neutralising IgY, while other vaccines failed to yield a strong humoral immune response against EBOV GP. Besides, the rVSV vaccine has been widely used (> 40 000 recipients) to prevent the current Ebola outbreak in the DRC [51]. The Ebola vaccine candidates reported earlier have verified its immunogenicity in rodents and primates, but due to the differences in the immune systems of poultry and mammals, these studies cannot be used in direct guide immunisation in poultry. The immunogenicity of these vaccines was confirmed on laying hens, proving that there is a big difference in the immunogenicity of different vaccines on hens. An efficient immunisation protocol was subsequently designed, and high titer neutralising IgY antibody was prepared. To further confirm the protective efficacy of the anti-EBOV IgY, it needed to be verified in an EBOV challenge model EBOV can only be worked with in BSL4 containment. Due to limited conditions, live EBOV could not be operated on. An alternative was found, and high dose rVSV expressing EBOV GP was injected into newborn mice. Mice injected with rVSV achieved 100% death, while the control group injected with the same amount of PBS all survived; this phenomenon is limited to mice within 3 days of birth. 1-week-old mice have been injected with even higher doses of rVSV and did not die, suggesting that the infection model is limited to newborn mice. For proper dosage and schedule of IgY, at present, the optimal dosage and a plan for the use of heterologous antibody therapy, especially the treatment of avian antibody, is uncertain, and many parameters may affect such a schedule, especially the number of viruses to be neutralised. Taking into account the excessive usage of the challenge dose and the uncertainty of the heterologous antibody, multiple administrations were chosen in the passive protection model. This animal model was used to evaluate our antibodies and proved that treatment with high-concentration IgY at 2 and 24 hours after challenge can protect mice from death, thus proving IgY prepared in this study could effectively protect mice from a lethal dose of virus attack/load.

Antibody therapy played a critical role in antiviral endeavours over the past century. Passive immunisation was employed in treating a multitude of infectious diseases. In the West Africa Ebola outbreak, recent evidence suggests therapeutic antibody treatment post-exposure can affect the progression of EVD. The production of recombinantly manufactured mAbs can be clinically relevant. Therefore, antibody-based treatments should be further investigated for use in humans [12, 19, 22, 23, 52, 53]. The PAmoja TuLinde Maisha (PALM [together save lives]) clinical trials have been conducted in DRC since 2018. Three antibody-based treatments (mAb114, REGN-EB3 and ZMapp) were used to evaluate the therapeutic effect of EVD, and the results proved that the antibodies could significantly reduce EVD mortality. REGN-EB3 subsequently became the first FDA-approved treatment for Zaire EBOV infection [24]. Given the economic and medical restrictions in Africa, all the antibody therapeutic preparations are hindered by high cost, long preparation periods, and strict requirements for cold chain transportation conditions. These factors suggest there is an ongoing need for more sustainable and reliable EBOV therapeutic reagents. Poultry antibodies have the advantages of safety, high efficiency, stability, easy scale production and low costs, which makes it an excellent candidate for global therapeutic use in times of outbreak.

In this study, the avian antibodies platform was used to develop an EBOV therapeutic formulation based on poultry IgY, which was shown to be effective when administered as a post-exposure prophylactic in newborn Balb/c mice challenged with VSVΔG/EBOVGP. The conversion of antibody products derived from eggs into clinical practice is a considerable challenge. However, ongoing Phase III clinical trials have tested the avian IgY against Pseudomonas aeruginosa with the support of the European Medicines Agency [48, 54]. The efficacy of antibodies in the treatment of cystic fibrosis suggests that new methods based on avian antibodies can be used to develop therapies for prevention and treatment. IgY antibody-based treatments have promising research prospects. When compared with other mammalian antibodies, our anti-EBOV IgY antibody has the advantage of greatly improved thermal stability, lower cost mass-production and avoiding ADE reaction, therefore making it more suit for implementation in Africa. Ebola epidemics occur in impoverished, hot African areas where access to electricity and cold-chain storage is scarce. Current mammalian antibodies have limited clinical application due to low thermal stability. Due to excellent thermal stability, anti-EBOV IgY developed in this study provides a promising strategy to solve the current application problems of Ebola antibody-based treatments in Africa.

## Materials and methods

### Ethics statement

All animal experiments were approved by the Committee on the Ethics of Animal Experiments of the Institute of Microbiology, Chinese Academy of Sciences (IMCAS), and conducted in compliance with the recommendations in the Guide for the Care and Use of Laboratory Animals of the IMCAS Ethics Committee.

### Cells and animals

293T, Vero and BHK-21 cells were grown in Dulbecco’s modified Eagle’s medium (DMEM) supplemented with 10% fetal bovine serum and 100 U/mL penicillin-streptomycin (Gibco, Grand Island, NY, USA) at 37°C in 5% CO_2_. Sf9 insect cells were cultured in SF900 serum-free media at 28°C CO_2_- free incubator.

Pregnant Balb/c mice and guinea pigs were purchased from a commercial supplier (Charles River). Laying hens were purchased from Beijing Vital River Laboratory Animal Technology Co., Ltd. All animals were kept in sterile, autoclaved cages and provided with sterilised food and water.

### Generation of immunogens

Five EBOV immunogens based on different platforms were prepared as described previously [55–59], with some modification. All immunogens are designed based on EBOV GP of the 2014 Ebola Makona epidemic strain[60]. Briefly, EBOV GP gene sequence was inserted an additional A residue at position 1019 to 1025 results in a frameshift. Thus the complete GP could be expressed. The gene was subsequently optimised for enhanced transgene expression in mammalian cells and then produced synthetically. As a means to obtain a DNA vaccine, the GP coding sequence was cloned into a mammalian expression plasmid pCAGGS, transgene expression was verified by western blot. Recombinant EBOVGP (rEBOVGP) protein containing a C-terminal His-tag was obtained through using the insect baculovirus expression system. The EBOV GP gene was first cloned into the pFastBac vector, and the constructed plasmid was transformed into *E*.*coli* DH10Bac cells to generate a recombinant bacmid. Then Sf9 cells were transfected with recombinant bacmid to generate recombinant baculovirus. After three consecutive passages, the cell culture supernatants were harvested and purified by Ni-NTA affinity chromatography (GE Healthcare, USA). Similar to the preparation of rEBOVGP, to obtain EBOV-VLP, pFastBac Dual vector including EBOV GP and VP40 genes was transformed into DH10Bac, and the resulting bacmid was transfected into Sf9 cells to generate recombinant baculovirus co-expressing EBOV GP and VP40. Culture supernatants were clarified and then pelleted by ultracentrifugation at 30 000 × g for 1 h at 4°C. The pellets were subsequently resuspended in PBS and further purified through a 10–50% (w/v) discontinuous sucrose gradient at 25 000 × g for 1.5 h at 4°C. The visible band between 30% and 50% density range was collected and re-suspended in PBS. The resulting protein products are EBOV-VLP. A recombinant E1/E3-deleted adenovirus type-5 vector-based EBOV vaccine was created by displacing of adenovirus E1 gene with EBOVGP, forming a recombinant Ad5/EBOVGP genome. The Ad5/EBOVGP was rescued by transfecting the genome into 293T cells and further propagated and purified by CsCl density gradient centrifugation. The number of virus particles (vp) was determined using optical density (260 nm) measurement. The resulting Ad5/EBOVGP could not replicate inside human tissues. To engineer the recombinant vesicular stomatitis virus (VSV) expressing EBOVGP, the VSVGP gene in rVSV replicon vector pVSV-XN2 was replaced by EBOVGP to generate pVSV-XN2/EBOVGP. The recombinant VSVΔG/EBOVGP was subsequently recovered using reverse genetics by co-transfecting pVSV-XN2/ EBOVGP and pBluescript SK+ (pBS) plasmids expressing the VSV nucleocapsid (N), phosphoprotein (P) and large polymerase subunit (L) into BHK-21 cells. rVSV virions were plaque purified, and virus titers were determined by standard plaque assay using BHK-21 cells. All EBOV immunogens were verified by western blot.

### Immunizations

Seven groups of 5-month-old laying hens (n = 5 per group) were inoculated i.m with immunogens prepared as described above. The detailed scheme is 10^3^ or 10^4^ TCID50 VSVΔG/EBOVGP, 100 μg rEBOVGP, 100 μg pCAGGS/EBOVGP, 10 μg EBOV-VLP, 10^11^ VP Ad5/EBOVGP, or an equivalent volume of PBS as a sham control at weeks 0, 2, 4, 6 (4 times). Eggs were collected at weeks, 0, 2, 4, 6, 8 for ELISA and NAbs test.

### Purification of yolk IgY antibody

IgY was isolated from the egg yolk using the water dilution method, a fast and straightforward way was used to separate IgY from the yolk. The separation method is improved based on previous research. Yolks were isolated, the yolk membrane was discarded, and the contents were diluted 1:8 with cold de-ionised water. Dilution was stirred uniformly, acidified to pH 5.0, keeping stability for a period of 10 h then centrifuged at 10 000 × g for 15 min, and the supernatant was garnered. To further purify the IgY antibody, 1% (by volume) of the solution of n-octanoic acid was slowly added with stirring to the supernatant and was centrifuged and filtered in the same way. Finally, The IgY was further purified by gel filtration (Superdex 200, GE Healthcare), eluted in PBS (pH 7.2) buffer, and concentrated by ultrafiltration to approximately 20 mg/ml using 100-kDa cut-off membranes (Millipore).

### SDS–PAGE and Western blot analysis

Sodium dodecyl sulphate-polyacrylamide gel electrophoresis (SDS–PAGE) was performed to determine the purity of IgY. The samples were mixed with reducing sample buffer, heated at 98°C for 10 min. Ten microliters of the sample were subsequently loaded into each well to SDS-PAGE (12% gel, staining for 3 h and destaining for 2 h). The pre-stained protein standard (Fermentas, Lithuania) was used as a molecular weight marker. The protein bands were visualised with Coomassie Brilliant Blue R250 (Fluka USA). The gel was analysed using Bio-Rad image analysis software.

Western blot was performed to check the specificity of the prepared immunogens. VSVΔG/EBOVGP, rEBOVGP, EBOV-VLP, Ad5/EBOVGP and cell lysate transfected with pCAGGS/EBOVGP plasmid were separated using SDS-PAGE on 15% polyacrylamide gels. For Western blot analysis, the proteins were electrically transferred onto a polyvinylidene difluoride (PVDF) membrane using a semi-dry blotting apparatus (15V, 40 min, RT), then blocked with Tris-buffered saline containing 0.05% Tween 20 (TBS-T) and 5% non-fat dry milk for 1 h at RT and was incubated overnight at 4°C with a 1:5000 dilution of mouse anti-EBOVGP1 specific mAb (R&D systems, Clone # 993408, USA). After having been washed five times with TBS-T, the membrane was incubated with HRP-conjugated goat anti-mouse IgG diluted 1:2000 (Promega, Madison, Wisconsin, USA) for 2 h at RT. The membrane was washed 4 times. Specific binding bands were then detected by incubation in substrate buffer containing 4 mg 3,3′-diaminobenzidine tetrahydrochloride (Aladdin, China) in 5 mL Tris–HCl and 15 μl hydrogen peroxide for 3–5 min. This reaction was stopped by rinsing with distilled water.

### ELISA

EBOVGP-specific ELISA was used to determine endpoint binding antibody titers of immune yolk IgY. Endpoint titers were defined as the reciprocal serum dilution that yielded an OD450 > 2-fold over background values. 96-well plates were briefly coated with 10 μg/mL rEBOVGP in a carbonate-bicarbonate buffer, pH 9.6, at 4°C overnight. The plates were then blocked with 5% skim milk in PBS (pH 7.4) at 37°C for 1 h. IgY was added to the top row (1:40), and 2-fold serial dilutions were tested in the remaining rows. The plates were incubated at 37°C for 1 h, followed by five washes with PBST. Subsequently, the plates were incubated with 100 μl of HRP-conjugated rabbit anti-chicken IgY working solution at 37°C for 30 min and washed with PBST five times. The assay was developed using 3,3’,5’,5-Tetramethylbenzidine HRP substrate (TMB) with 100 μl each well stopped by the addition of 50 μl of 2 M H_2_SO_4_ for 10 min. Plates were measured at 450nm by a microplate reader using Softmax Pro 6.0 software (Molecular Devices, CA, USA). PBS was used as a blank control. Simultaneously a negative control (IgY derived from PBS-immunized hens) was ascertained in each plate. All ELISA measurements were repeated at least three times with each sample in triplicate.

### Pseudotyped virus neutralisation assay

Two pseudoneutralisation (PsN) assays were performed, based on non-replicating VSV pseudotype and lentiviral pseudotype, respectively. EBOV GP pseudotyped lentiviral and VSV virions with a luciferase reporter were produced as previously described [61, 62]. The resulting lentiviral particle could achieve a single-round infection. In brief, Vero cells were plated in 96-well plates and cultured overnight. IgY serial dilutions (1:10, 1:20, 1:40, etc., in DMEM) and pseudo particles were mixed in a ratio of 1: 9 and incubated at 37°C for 1 h, before addition to pre-plated target cells in 96-well culture plates (density of 10^4^ cells/well) with 3 replicates. The luciferase activities of infected cells were examined 36 h post-infection. Sample dilutions which showed a 50% reduction in the number of fluorescing cells compared to controls were considered to NAb titers.

### Adoptive transfer experiment

The yolk was collected and purified two weeks after the final vaccination. Eight groups of newborn BALB/c mice within three days (n = 5 per group) were challenged by the s.c. route with 10^4^ TCID_50_ VSVΔG/EBOVGP, after 2 or 24 h, challenged mice were treated i.p. with 10^4^, 10^5^, or 10^6^ NAU/kg anti-EBOV IgY twice daily for 3 days, respectively. Clinical symptoms, bodyweight change rate and survival rate were monitored within 15 days.

### Metabolic studies in guinea pigs

Two Groups of guinea pigs (n = 5 per group) were administrated s.c. with 10^6^ or 10^5^ NAU/kg purified anti-EBOV IgY. Sera were collected daily within 6 days for NAb titers determination.

### Statistical analysis

GraphPad Prism software (GraphPad, Inc., San Diego, CA) was used to graph and to conduct statistical comparisons of all data as described in the text. Results were presented as the mean± standard deviation unless indicated separately. The one-way or two-way analysis of variance followed by Tukey–Kramer multiple comparison test, two-sided log-rank tests, Tukey–Kramer multiple comparison tests were used in some experiments. *P < 0.05, significant; ** *P* < 0.01, very significant; *** *P* < 0.001, highly significant; **** *P* < 0.0001, extremely significant, ns *P* > 0.05, not significant. Investigators were not blinded for data collection or analysis.
